# Investigating the response scale of the EORTC QLQ-C30 in German cancer patients and a population survey

**DOI:** 10.1186/s12955-021-01866-x

**Published:** 2021-10-09

**Authors:** Michael Koller, Karolina Müller, Sandra Nolte, Heike Schmidt, Christina Harvey, Ulrike Mölle, Andreas Boehm, Daniel Engeler, Jürg Metzger, Monika Sztankay, Bernhard Holzner, Mogens Groenvold, Dagmara Kuliś, Andrew Bottomley, the EORTC Quality of Life Group

**Affiliations:** 1 Center for Clinical Studies, University Hospital Regensburg, 93042 Regensburg, Germany; 2 Division of Psychosomatic Medicine Berlin, Charité – Universitätsmedizin Berlin, Berlin, Germany; 3 Institute of Health and Nursing Science, Martin Luther University, Halle-Wittenberg, Halle (Saale), Germany; 4 Department for Radiation Medicine University Clinic and Outpatient Clinic for Radiotherapy, University Hospital Halle (Saale), Halle (Saale), Germany; 5 St. Marienwörth Hospital, Bad Kreuznach, Germany; 6 Department of Otolaryngology Head and Neck Surgery, St. Georg Hospital, Leipzig, Germany; 7 Department of Urology, Cantonal Hospital St. Gallen, St. Gallen, Switzerland; 8 Department of General Surgery, Cantonal Hospital Lucerne, Luzern, Switzerland; 9 Department of Psychiatry, Psychotherapy and Psychosomatics, Medical University of Innsbruck, and University Hospital of Psychiatry II, Innsbruck, Austria; 10 Palliative Care Research Unit, Department of Geriatrics and Palliative Medicine, Bispebjerg and Frederiksberg Hospital, University of Copenhagen, Copenhagen, Denmark; 11 Quality of Life Department, EORTC Headquarters, Brussels, Belgium

**Keywords:** Quality-of-life, Patient-reported outcomes, Response scales, Responder behaviour, Cognitive processes

## Abstract

**Background:**

The European Organization for research and Treatment of Cancer (EORTC) Core Quality of Life Questionnaire (QLQ-C30) scales are scored on a 4-point response scale, ranging from *not at all* to *very much*. Previous studies have shown that the German translation of the response option *quite a bit* as *mäßig* violates interval scale assumptions, and that *ziemlich* is a more appropriate translation. The present studies investigated differences between the two questionnaire versions.

**Methods:**

The first study employed a balanced cross-over design and included 450 patients with different types of cancer from three German-speaking countries. The second study was a representative survey in Germany including 2033 respondents. The main analyses included compared the *ziemlich* and *mäßig* version of the questionnaire using analyses of covariance adjusted for sex, age, and health burden.

**Results:**

In accordance with our hypothesis, the adjusted summary score was lower in the *mäßig* than in the *ziemlich* version; Study 1: − 4.5 (95% CI − 7.8 to − 1.3), *p* = 0.006, Study 2: − 3.1 (95% CI − 4.6 to − 1.5), *p* < 0.001. In both studies, this effect was pronounced in respondents with a higher health burden; Study 1: − 6.8 (95% CI − 12.2 to − 1.4), *p* = 0.013; Study 2: − 4.5 (95% CI − 7.3 to − 1.7), *p* = 0.002.

**Conclusions:**

We found subtle but consistent differences between the two questionnaire versions. We recommend to use the optimized response option for the EORTC QLQ-C30 as well as for all other German modules.

*Trial registration*: The study was retrospectively registered on the German Registry for Clinical Studies (reference number DRKS00012759, 04th August 2017, https://www.drks.de/DRKS00012759).

**Supplementary Information:**

The online version contains supplementary material available at 10.1186/s12955-021-01866-x.

## Background

The European Organization for Research and Treatment of Cancer Core Quality of Life Questionnaire (EORTC QLQ-C30) is a 30-item questionnaire and 28 out of 30 items are scored on a 4-point Likert response scale: 1 = *not at all*, 2 = *a little*, 3 = *quite a bit*, and 4 = *very much* [1]. The German equivalents have been translated as 1 = *überhaupt nicht, 2* = *wenig, 3* = *mäßig, and 4* = *sehr* [2]. Ideally, multi-item Likert scales should be interval scaled, which assumes equidistance between response options.

Research suggests that the German wording of the EORTC QLQ-C30 response scale, particularly the term *mäßig* for response category 3 (which in English is supposed to stand for *quite a bit*), may not be optimal [3, 4]. Based on these findings, we conducted three studies involving students, cancer patients, and adult control subjects (total number of participants N = 334) to investigate the intensity rating of the critical term *mäßig* relative to intensity ratings of other terms that seemed to be more appropriate for response category 3, such as *einigermaßen, überwiegend* or *ziemlich*. The task of the research participants was to rate each term on a 0–100 linear intensity scale (with the anchors 0 = *überhaupt nicht* [*not at all*] and 100 = *sehr* [*very much*]). The currently used term *mäßig* yielded an average intensity rating of 42 and thus, was rated substantially lower than the ideal value of 67 (difference − 25). In contrast, *ziemlich* turned out to be the best choice for response category 3, with mean intensity rating of 71, and it was among the top three terms for response option “3” in each study (see Additional file 1: Appendix Table S1).

Research undertaken by Schwarz and Strack [5, 6] showed that response scales influence respondents’ answers to questions. For example, respondents consistently reported higher frequencies for certain response options on scales with high rather than low frequency response alternatives [5]. Following this logic, we assumed that changes in the current German response format of the EORTC QLQ-C30 items will lead to changes in reported symptom and functioning scores. If *mäßig* is semantically very close to *wenig* (in English *a little*), it does not constitute a reasonable response alternative for patients with a moderate/considerable health problem. They might then tend to skip *mäßig* and turn to the next higher response alternative *sehr* (in English *very much*). Thus, we hypothesized that the current German response scale of the EORTC QLQ-C30 (*mäßig* version) leads to higher symptom scores and lower functioning scores than an optimized version (*ziemlich* version) with a category-label 3 that is equidistant between response categories 2 and 4. This effect should be particularly pronounced in patients with considerable health problems. The present pair of studies were designed to test this hypothesis.

## Methods

### Study 1

#### Study design and sample size rationale

This study involved patients with different types of cancer. It was a randomized cross-over-design study allowing for within-subject comparisons of the current and updated questionnaire versions. Patients were randomized either to a paper-based or a tablet-based version of the EORTC QLQ-C30 (see Additional file 1: Appendix Figure S1). A commonly accepted rule of thumb recommends a ratio of 10–15 respondents per item [7]. Given that the EORTC QLQ-C30 compromises 30 items, a sample size of 300–450 respondents is adequate. Data were collected between April 2016 and September 2018 at 7 study sites in Germany, Austria, and Switzerland.

#### Ethical considerations

The study was approved by the Ethical Committee of the University of Regensburg (reference number 14-101-0209) and by local ethical committees of the other study sites. The study was registered on the German Registry for Clinical Studies (reference number DRKS00012759), which is part of the WHO Trial Registration Data Set.

#### Inclusion and exclusion criteria

Inclusion criteria were histologically confirmed diagnosis of cancer, mentally and physically fit to complete a questionnaire, able to understand German, 18 years of age or above (no upper age limit), and informed consent. Patients who were mentally and physically unfit to complete a questionnaire or denied informed consent were excluded.

#### Procedure

Patients were approached by a researcher and subsequently informed about the study. After providing written informed consent, patients were randomly assigned to the paper-based or computer-based assessment. The paper version involved the standard two-page EORTC QLQ-C30 questionnaire, in which the response options are numbered from 1 to 4 for each item of the questionnaire, with the appropriate labels appearing at the top of each section. In the electronic version [8], each item is presented separately on screen together with the response options. Regardless of paper version or electronic version, patients were randomly assigned to fill in the questionnaire using conventional German response options (i.e., *überhaupt nicht, wenig, mäßig, sehr)* of the EORTC QLQ-C30 version 3.0 or using the optimized version in which *mäßig* was replaced by *ziemlich*. Patients filled in the questionnaire again at a later point in time, whereby the alternate response option version was presented, and continued with either paper-based or computer-based assessment depending on the assigned study arm. Additionally, patients rated on two anchor variables whether their health/QoL improved, worsened, or remained unchanged between both assessments to ensure that differences between EORTC QLQ-C30 versions within a patient is attributed to questionnaire versions and not real changes in health/QoL.

### Study 2

#### Study design and sample size rationale

The data were collected in 13 European countries, the USA and Canada in the context of an international project to generate European general population norm data for the EORTC QLQ-C30 questionnaire [9, 10]. Sample size per country was based on the following rationale: stratification by sex and age groups (18–39, 40–49, 50–59, 60–69, 70 + years), with a target sample size of each sex x age x country subgroup of n = 100, leading to an anticipated sample size of n = 1000/country. This sampling design was considered sufficient to investigate differential item functioning (DIF) using logistic regression analysis which was at the core of the original study [10]. Data collection was performed by GfK SE (www.gfk.com), a panel research company specialized in representative multinational online surveys. Panel members register voluntarily and generally participate when contacted, resulting in response rates between 75 and 90% [9]. Data were collected in March/April 2017. German respondents were randomly assigned either to the conventional EORTC QLQ-C30 questionnaire version 3.0 (response option 3 = *mäßig*, *n* = 1006) or the optimized version (response option 3 = *ziemlich*, *n* = 1027).

#### Ethical considerations

The multinational survey conformed to the common ethical standards by obtaining informed consent from all participants before collecting data completely anonymously. Any identification of the respondents through the authors is impossible. The study thus complies with the EU General Data Protection Regulations as well as with the professional standards of the European Pharmaceutical Market Research Association (EphMRA), which GfK SE is a member of.

#### Inclusion and exclusion criteria for the present analyses

Respondents were eligible if they provided informed consent. Since these were all registered panel members, all persons contacted were able to read and understand a sufficient level of German and they also had access to a computer, as data collection was done electronically. For the present analyses only respondents from Germany were used.

#### Procedure

Subjects were contacted by the survey company GfK SE. Samples were stratified with an equal number of men and women, and 5 pre-defined age categories, i.e., 18–39 years, 40–49, 50–59, 60–69, and 70 years and above, resulting in *n* = 200 per age/sex stratum. As part of the online panel, respondents were asked to complete the 30 items of the EORTC QLQ-C30 [10]. Comparable to study 1, each item was presented separately on screen.

### Statistical analyses

EORTC QLQ-C30 scales were computed according to the EORTC Scoring Manual [11]. In a first step, all scales were linearly transformed (0–100), so that for the five functioning scales, higher scores represent higher functioning and for the nine symptom scales, higher scores represent higher symptom burden. In a second step, a summary score was calculated, consisting of 13 out of the 15 scales, excluding financial difficulties and global health status/quality-of-life. For this summary score, the symptom scales were reversed, so that 0 represents lowest and 100 highest QoL [12].

We employed the following strategy in using and interpreting scale results: we first had a look at the statistically significant difference (*p* value < 0.05) in the summary score. If a significant difference was obtained, we inspected significant differences with regard to the 14 single symptom or functioning scales. This strategy was chosen in order to address multiplicity issues. To determine clinically meaningful differences we used the conservative 5 point criterion (small difference) [13].

The core analyses related to differences between the conventional EORTC QLQ-C30 version (*mäßig*) and the optimized EORTC QLQ-C30 version (*ziemlich*) and included univariable analyses of the unadjusted means (*t* tests) as well as multivariable analyses. More specifically, two separate analyses were conducted on the cancer patient sample: between-subject and within-subject comparisons. For between-subject comparisons, responses to both questionnaire versions of the first assessment were compared using analyses of covariance (ANCOVAs) adjusted for sex, age, mode of administration (MOA, paper vs. electronic), and health burden. Health burden was defined by the EORTC QLQ-C30 scale global quality-of-life: < 50 (worse QoL) vs. ≥ 50 (better QoL) [14, 15].

For within-subject comparisons, mixed linear models were used: subject as random factor, questionnaire version as repeated factor and the following set of fixed factors: questionnaire version, MOA, order of questionnaire versions, sex, age, and health burden. The mixed linear models included only patients who reported no changes in QoL and health between both assessments on the two anchor questions.

In the German population sample, differences between the two EORTC QLQ-C30 versions were assessed using ANCOVAs adjusted for sex, age, and health burden.

Parametric methods were used for all analyses due to their robustness to violations of non-normality, which is occasionally the case with QoL data [16].

Furthermore, according to classical test theory, basic psychometric performance (internal consistency [17] as well as convergent and discriminant validity [18, 19]) of both EORTC QLQ-C30 versions were explored (see Additional file 1: Appendix Basic psychometric properties and Table S2).

Statistical analyses were carried out using SPSS 25. Statistical tests were two-sided and were done at the 0.05 significance level. Descriptive statistics included the following: frequencies (*n*), percentages (%), means (m), standard deviations (sd), 95% confidence intervals (CI), medians (med), interquartile ranges (IQR).

## Results

### Study 1

In total, 467 patients were recruited. Seventeen patients were excluded from analyses due to the following reasons: physically or mentally unfit (n = 10), declined participation during first assessment (n = 5), and study data were overwritten due to technical issues (n = 2). Thus, data of 450 patients (median age = 63 years, 46% females) were available (Table 1). A second assessment could be obtained in 404/450 patients (90%), which is a high completion rate for second assessment [20]. The median gap between the two assessment points was 4 days (IQR = 2/7) (Additional file 1: Appendix Figure S1). Accidently, four patients responded twice to the same questionnaire version and had to be excluded for test–retest analyses.

**Table 1:** Study 1: patient characteristics

Cancer patients (*N* = 450)	
Age in years m (SD); med (IQR); min–max	62.2 (12.3), 63.0 (54.0–71.0), 21–89
Sex no. (%)	
Female	209 (46.4)
Male	241 (53.6)
Education no. (%)	
Less than some post compulsory education	176 (39.1)
At least some post compulsory (~ upper secondary) education	272 (60.4)
Missing	2 (0.4)
Country no. (%)	
Germany	393 (87.3)
Switzerland	37 (8.2)
Austria	20 (4.4)
Cancer^a^ no. (%)	
Oral cavity and throat (C00–C14)	32 (7.1)
Digestive organs (C15–C26)	93 (20.7)
Respiratory and chest organs (C30–C39)	50 (11.1)
Bones and joint cartilage (C40–C41)	2 (0.4)
Malignant melanoma (C43)	37 (8.2)
Non-melanoma skin cancer (C44)	26 (5.8)
Skin cancer not defined (C43–C44)	5 (1.1)
Soft and mesothelial tissue (C45–C49)	2 (0.4)
Mammary gland (C50)	45 (10.0)
Female sex organs (C51–C58)	29 (6.4)
Male sex organs (C60–C63)	41 (9.1)
Urinary organs (C64–C68)	29 (6.4)
Eye, brain, and central nervous system (C69–C72)	4 (0.9)
Endocrine glands (C73–C75)	3 (0.7)
Cancer of unknown primary syndrome (C76–C80)	9 (2.0)
Blood and lymph gland cancer (C81–C96)	36 (8.0)
Pituitary adenoma (D35)	1 (0.2)
More than one diagnosis	5 (1.1)
Unknown	1 (0.2)
Time from initial cancer diagnosis to initial assessment^b^ in months m (SD); med (IQR); min–max	25.9 (52.6), 4.6 (1.6–21.1), 0–366
Cancer stage no. (%)	
Local	176 (39.1)
Locally advanced	123 (27.3)
Metastatic	133 (29.6)
Missing/not applicable^c^	18 (4.0)
Hospitalization no. (%)	
Inpatient	291 (64.7)
Outpatient	159 (35.3)
Treatment status no. (%)	
Pretreatment	13 (2.9)
In treatment	387 (86.0)
Aftercare	50 (11.1)
Currently on treatment no. (%)	
No	63 (14.0)
Yes	387 (86.0)
Systemic treatment	152 (33.8)
Local treatment	168 (37.3)
Systemic and local treatment	67 (14.9)
Previous treatment no. (%)	
Unknown	111 (24.7)
No	169 (37.7)
Yes	170 (37.8)
Systemic treatment	23 (5.1)
Local treatment	99 (22.0)
Systemic and local treatment	48 (10.7)
Comorbidity no. (%)	
No	97 (21.6)
Yes at least one additional disease	353 (78.4)
Multiple answers possible (sum > 100%)	
Injuries	43 (9.6)
Diseases of the musculoskeletal system	103 (22.9)
Cardiovascular diseases	192 (42.7)
Respiratory diseases	79 (17.6)
Mental impairment	47 (10.4)
Neurological and sensory diseases	69 (15.3)
Diseases of the digestive system	84 (18.7)
Diseases of the urogenital tract	73 (16.2)
Skin diseases	50 (11.1)
Metabolic and hormonal disorders	129 (28.7)
Blood disorder	17 (3.8)
Congenital diseases	11 (2.4)
Other	6 (1.3)

*m* mean, *SD* standard deviation, *med* median, *IQR* inter quartile range, *systemic treatment* chemotherapy, hormone therapy, immunotherapy, stem cell transplantation, photopheresis, *local treatment* operation, radio therapy, high intensity focused ultrasound

^a^The primary cancer site was counted for metastatic cancer. *n* = 27 patients were previously diagnosed with another cancer type; the current cancer type was counted. In 8 cases, it was specified that the cancer relapsed and in 11 cases it was specified that the patient is currently cancer free

^b^One patient was excluded due to inconsistent data

^c^*n* = 16 malignant neoplasms of lymphoid, haematopoietic and related tissue

In the first step, we analyzed differences in EORTC QLQ-C30 scores between patients who received either the *mäßig* or *ziemlich* version at the first assessment. As shown in Table 2, the unadjusted analysis showed no significant differences in the summary score between the two questionnaire versions (mean = 70.1, sd = 19.9 vs. m = 73.0, sd = 18.6; *p* = 0.116). Multivariable analyses adjusted for age, sex, MOA, and health burden, showed a mean difference of − 4.5 (95% CI − 7.8 to − 1.3) in the summary score (*p* = 0.006), such that the *mäßig*-version yielded lower scores (poorer QoL) than the *ziemlich*-version (Table 3). Mean differences for all 14 scale scores were in the expected direction (i.e., higher symptoms and lower functioning in the mäßig- than in the ziemlich-version), with four showing a statistically significant difference (*p* values < 0.05) (Table 3), and all were > 5 score points.

**Table 2:** Comparisons between QLQ-C30 versions—univariable analyses (unadjusted)

	Cancer patients between-group comparisons *N* = 450^a^						Cancer patients within-group comparisons *N* = 229^b^					German population between-group comparisons *N* = 2033				
	*mäßig* version (*n* = 226)		*ziemlich* version (*n* = 224)			*mäßig* version (*n* = 229)			*ziemlich* version (*n* = 229)			*mäßig* version (*n* = 1006)		*ziemlich* version (*n* = 1027)		
	m	sd	m	sd	*p*	m		sd	m	sd	*p*	m	sd	m	sd	*p*
PF	70.5	26.5	69.5	25.0	0.674	73.3		25.3	77.3	21.5	< 0.001	82.0	21.5	84.2	19.7	0.019
RF	62.0	35.1	62.8	34.5	0.813	64.5		31.6	68.4	30.3	0.007	80.3	27.4	82.4	25.2	0.067
EF	61.0	27.5	67.4	23.6	0.008	68.9		24.7	71.6	22.5	0.008	75.1	24.2	76.1	23.0	0.318
CF	76.5	26.3	80.7	22.8	0.072	81.9		23.7	83.1	20.3	0.194	85.4	21.1	86.6	18.9	0.163
SF	62.5	33.7	65.9	30.8	0.257	67.8		31.4	70.1	29.1	0.111	85.1	25.5	86.7	22.9	0.144
FA	44.4	29.7	41.8	29.3	0.356	36.9		28.1	34.8	25.9	0.064	31.4	27.7	29.1	24.8	0.050
NV	12.2	20.0	11.9	21.0	0.892	7.2		14.1	7.5	16.1	0.772	5.2	15.7	4.5	14.0	0.284
PA	30.0	31.9	28.9	30.6	0.716	26.6		30.1	24.3	27.6	0.062	28.3	31.1	26.2	28.0	0.101
DY	28.0	32.5	27.2	31.7	0.806	25.9		32.4	23.7	28.9	0.108	19.6	27.8	17.8	25.3	0.109
SL	41.0	36.4	32.1	32.2	0.006	32.9		34.9	28.5	29.8	0.005	28.9	33.6	27.9	31.1	0.525
AP	30.1	35.3	26.1	34.6	0.230	23.0		33.1	20.0	28.2	0.058	9.3	22.2	7.9	19.3	0.123
CO	17.3	29.3	16.4	28.5	0.764	13.4		24.5	12.7	24.8	0.560	8.9	21.6	8.0	19.8	0.296
DI	18.0	29.3	15.8	25.9	0.410	13.1		24.4	12.7	22.5	0.746	9.7	22.2	8.0	19.3	0.067
FI	22.8	31.1	20.5	28.0	0.414	20.7		30.6	18.7	26.7	0.140	10.4	24.1	9.2	22.4	0.238
Summary	70.1	19.9	73.0	18.6	0.116	75.1		18.3	77.4	16.8	< 0.001	82.0	17.7	83.6	15.9	0.038

The EORTC QLQ-C30 was presented in two versions. The conventional version used *mäßig* and the optimized version used *ziemlich* as response option 3 of the 4-point Likert scale

*PF* physical functioning, *RF* role functioning, *EF* emotional functioning, *CF* cognitive functioning, *SF* social functioning, *FA* fatigue, *NV* nausea and vomiting, *PA* pain, *DY* dyspnea, *SL* insomnia, *AP* appetite loss, *CO* constipation, *DI* diarrhea, *FI* financial difficulties

^a^For the between-group comparisons, QLQ-C30 data of cancer patients of the first assessment were compared

^b^For the within-group comparisons, cancer patients without changes in health and quality of life between both assessments were used

**Table 3:** Comparisons between QLQ-C30 version—multivariable analyses (adjusted)

	Questionnaire version *mäßig – ziemlich*				Higher health burden (QoL < 50) *mäßig – ziemlich*				Lower health burden (QoL ≥ 50) *mäßig – ziemlich*				Paper− based assessment *mäßig – ziemlich*				Computer− based assessment *mäßig – ziemlich*			
	Delta	95% CI		*p*	Delta	95% CI		*p*	Delta	95% CI		*p*	Delta	95% CI		*p*	Delta	95% CI		*p*
Cancer patients between− group comparisons^a^																				
*n*	*450*				*144*				*306*				*236*				*214*			
PF	− 1.0	− 5.5	3.5	0.663	− 2.1	− 9.5	5.3	0.585	0.1	− 5.0	5.1	0.978	2.3	− 3.7	8.3	0.450	− 4.3	− 10.5	2.0	0.179
RF	− 1.3	− 7.4	4.8	0685	0.7	− 9.4	10.8	0.894	− 3.2	− 10.1	3.7	0.363	− 1.8	− 10.0	6.3	0.660	− 0.7	− 9.2	7.9	0.687
EF	− 7.5	− 12.1	− 2.8	0.002	− 8.8	− 16.4	− 1.1	0.024	− 6.2	− 11.4	− 0.9	0.022	− 6.6	− 12.8	− 0.4	0.038	− 8.4	− 14.8	− 1.9	0.011
CF	− 5.2	− 9.9	− 0.6	0.028	− 6.3	− 14.0	1.4	0.107	− 4.1	− 9.4	1.1	0.123	− 3.1	− 9.4	3.1	0.324	− 7.3	− 13.8	− 0.8	0.028
SF	− 4.9	− 10.7	0.9	0.095	− 7.4	− 16.9	2.2	0.130	− 2.5	− 9.0	4.1	0.459	− 5.5	− 13.2	2.3	0.167	− 4.4	− 12.5	3.7	0.287
FA	4.8	− 0.3	9.9	0.063	7.4	− 1.0	15.7	0.085	2.3	− 3.5	8.0	0.435	4.6	− 2.2	11.4	0.182	5.0	− 2.1	12.1	0.165
NV	1.1	− 2.8	5.1	0.571	2.5	− 4.0	9.0	0.444	− 0.3	− 4.7	4.2	0.908	0.1	− 5.1	5.4	0.959	2.1	− 3.4	7.6	0.446
PA	2.6	− 3.1	8.3	0.374	3.9	− 5.5	13.4	0.414	1.2	− 5.2	7.7	0.706	2.8	− 4.9	10.4	0.477	2.4	− 5.6	10.4	0.555
DY	1.2	− 4.9	7.3	0.700	− 0.6	− 10.7	9.4	0.901	3.0	− 3.9	10.0	0.390	− 3.2	− 11.4	5.0	0.446	5.6	− 3.0	14.1	0.200
SL	9.9	3.3	16.5	0.004	10.4	− 0.6	21.3	0.064	9.4	1.9	17.0	0.014	8.0	− 0.8	16.9	0.076	11.7	2.5	21.0	0.013
AP	7.4	1.0	13.7	0.024	13.1	2.5	23.7	0.015	1.6	− 5.6	8.8	0.664	5.9	− 2.7	14.5	0.177	8.8	− 0.1	17.7	0.053
CO	2.1	− 3.5	7.8	0.463	3.8	− 5.6	13.1	0.429	0.5	− 5.9	6.8	0.888	0.2	− 7.4	7.7	0.967	4.1	− 3.8	11.9	0.312
DI	5.3	− 0.3	10.6	0.062	12.4	3.5	21.4	0.007	− 2.1	− 8.3	4.0	0.491	3.6	− 3.7	10.8	0.332	6.7	− 0.8	14.3	0.082
FI	5.0	− 0.7	10.7	0.086	12.4	3.0	21.9	0.010	− 2.4	− 8.9	4.0	0.458	3.5	− 4.2	11.1	0.374	6.5	− 1.4	14.5	0.108
Summary	− 4.5	− 7.8	− 1.3	0.006	− 6.8	− 12.2	− 1.4	0.013	− 2.3	− 5.9	1.4	0.226	− 3.1	− 7.5	1.2	0.161	− 5.9	− 10.5	− 1.4	0.010
Cancer patients within—group comparisons^b^																				
*n*	*229*				*57*				*172*				*119*				*110*			
PF	− 4.5	− 6.4	− 2.6	< 0.001	− 5.4	− 8.7	− 2.2	0.001	− 3.6	− 5.4	− 1.7	< 0.001	− 4.0	− 6.4	− 1.6	0.001	− 5.0	− 7.5	− 2.4	< 0.001
RF	− 4.8	− 8.1	− 1.4	0.005	− 6.3	− 12.1	− 0.5	0.033	− 3.2	− 6.6	0.1	0.056	− 4.5	− 8.8	− 0.3	0.038	− 5.0	− 9.5	− 0.5	0.031
EF	− 2.5	− 4.8	− 0.3	0.026	− 2.5	− 6.4	1.4	0.209	− 2.6	− 4.8	− 0.4	0.023	− 2.6	− 5.4	0.3	0.077	− 2.5	− 5.5	0.6	0.109
CF	− 1.5	− 3.6	0.7	0.190	− 1.9	− 5.6	1.9	0.335	− 1.0	− 3.2	1.1	0.344	− 0.9	− 3.7	1.9	0.548	− 2.1	− 5.0	0.9	0.175
SF	− 4.1	− 7.3	− 0.9	0.012	− 7.9	− 13.5	− 2.3	0.006	− 0.4	− 3.6	2.8	0.825	− 4.4	− 8.5	− 0.2	0.038	− 3.9	− 8.3	0.5	0.081
FA	3.4	0.9	6.0	0.009	6.1	1.6	10.6	0.008	0.8	− 1.8	3.4	0.537	3.1	− 0.2	6.4	0.064	3.8	0.3	7.3	0.036
NV	0.4	− 1.9	2.7	0.708	1.9	− 2.1	5.9	0.356	− 1.0	− 3.3	1.3	0.392	1.0	− 1.9	4.0	0.486	− 0.2	− 3.3	3.0	0.915
PA	3.3	0.5	6.2	0.021	5.3	0.4	10.2	0.035	1.4	− 1.4	4.2	0.335	1.6	− 2.0	5.3	0.379	5.1	1.2	8.9	0.010
DY	4.0	1.0	7.0	0.010	7.4	2.1	12.7	0.006	0.6	− 2.4	3.7	0.691	1.0	− 2.9	4.9	0.614	7.0	2.8	11.2	0.001
SL	4.7	1.0	8.5	0.014	5.2	− 1.3	11.8	0.118	4.3	0.5	8.1	0.026	3.6	− 1.2	8.5	0.143	5.9	0.8	11.0	0.025
AP	4.9	1.3	8.5	0.008	8.2	1.9	14.6	0.011	1.5	− 2.1	5.2	0.403	2.3	− 2.4	7.0	0.329	7.5	2.5	12.4	0.003
CO	1.2	− 1.6	4.1	0.403	2.0	− 2.9	7.0	0.416	0.4	− 2.5	3.2	0.794	− 0.8	− 4.4	2.9	0.674	3.2	− 0.7	7.1	0.105
DI	1.2	− 1.9	4.3	0.444	2.9	− 2.4	8.2	0.283	− 0.5	− 3.6	2.5	0.736	2.6	− 1.4	6.5	0.197	− 0.2	− 4.4	4.0	0.924
FI	3.2	0.0	6.3	0.049	5.3	− 0.2	10.8	0.058	1.0	− 2.1	4.2	0.517	2.2	− 1.9	6.2	0.290	4.2	− 0.1	8.5	0.058
Summary	− 3.1	− 4.3	− 1.9	< 0.001	− 4.8	− 6.9	− 2.8	< 0.001	− 1.4	− 2.6	− 0.2	0.022	− 2.4	− 3.9	− 0.8	0.003	− 3.9	− 5.5	− 2.3	< 0.001
German population between—group comparisons^c^																				
*n*	*2033*				*370*				*1663*											
PF	− 3.8	− 5.9	− 1.8	< 0.001	− 5.6	− 9.3	− 2.0	0.003	− 2.1	− 3.8	− 0.3	0.020								
RF	− 4.4	− 6.9	− 1.9	0.001	− 6.8	− 11.3	− 2.3	0.003	− 2.1	− 4.2	0.1	0.056								
EF	− 2.0	− 4.4	0.3	0.091	− 2.5	− 6.8	1.7	0.247	− 1.5	− 3.5	0.5	0.130								
CF	− 2.7	− 4.8	− 0.7	0.010	− 4.4	− 8.1	− 0.6	0.024	− 1.1	− 2.9	0.7	0.218								
SF	− 3.9	− 6.1	− 1.7	0.001	− 6.3	− 10.3	− 2.3	0.002	− 1.5	− 3.4	0.4	0.117								
FA	4.3	1.8	6.8	0.001	6.3	1.7	10.8	0.007	2.4	0.3	4.5	0.027								
NV	1.4	− 0.2	3.0	0.089	2.2	− 0.8	5.1	0.146	0.6	− 0.8	2.0	0.367								
PA	3.6	0.7	6.4	0.015	4.5	− 0.7	9.7	0.093	2.7	0.2	5.1	0.033								
DY	3.8	1.1	6.6	0.007	6.0	1.0	11.0	0.019	1.7	− 0.7	4.0	0.162								
SL	3.0	− 0.3	6.3	0.074	5.2	− 0.8	11.2	0.091	0.9	− 2.0	3.7	0.546								
AP	3.5	1.3	5.6	0.002	5.9	2.0	9.9	0.003	1.0	− 0.9	2.8	0.310								
CO	1.5	− 0.7	3.8	0.187	1.9	− 2.2	6.0	0.367	1.2	− 0.8	3.1	0.235								
DI	1.5	− 0.8	3.8	0.187	0.9	− 3.3	5.0	0.676	2.2	0.2	4.2	0.027								
FI	3.3	0.9	5.7	0.007	5.6	1.3	9.9	0.011	1.0	− 1.1	3.0	0.347								
Summary	− 3.1	− 4.6	− 1.5	< 0.001	− 4.5	− 7.3	− 1.7	0.002	− 1.6	− 2.9	− 0.3	0.016								

The EORTC QLQ-C30 was presented in two versions. The conventional version used *mäßig* and the optimized version used *ziemlich* as response option 3 of the 4-point Likert scale

*PF* physical functioning, *RF* role functioning, *EF* emotional functioning, *CF* cognitive functioning, *SF* social functioning, *FA* fatigue, *NV* nausea and vomiting, *PA* pain, *DY* dyspnea, *SL* insomnia, *AP* appetite loss, *CO* constipation, *DI* diarrhea, *FI* financial difficulties, *delta* mean difference

^a^For between-subject comparisons, responses to the current and updated questionnaire versions of the first assessment were compared using ANCOVAs adjusted for sex, age, mode of administration (MOA) and health burden

^b^For within-subject comparisons, mixed linear models were used: subject as random factor, questionnaire version as repeated factor and the following set of fixed factors: questionnaire version, mode of administration (MOA), order of questionnaire versions, sex, age, and health burden

^c^For between-subject comparisons, responses to the current and updated questionnaire versions were compared using ANCOVAs adjusted for sex, age, and health burden

When taking a closer look at patients with considerable health burden (global QoL < 50 points, *n* = 144), the differences between the *mäßig* and *ziemlich* versions became particularly pronounced, i.e., the mean difference in the summary score was − 6.8 (95% CI − 12.2 to − 1.4, *p* = 0.013), whereas it was only − 2.3 (95% CI − 5.9 to 1.4, *p* = 0.226) in patients with lower/no health burden (global QoL ≥ 50 score points, *n* = 306; Table 3). In addition, four of the 14 single scales of patients with higher health burden yielded statistically significant differences. The four single scales as well as the total score were > 5 score points.

The next step were within-group comparisons in patients who did not indicate a change in their health and QoL between assessments (*n* = 229). Univariable analyses showed a lower summary score in the *mäßig* (m = 75.1, sd = 18.3) than *ziemlich* version (m = 77.4, sd = 16.8; *p* < 0.001, Table 2). Furthermore, we observed corresponding statistically significant mean differences in four of the 14 single scales (*p* values < 0.05); however, none was > 5 points.

In multivariable analyses (Table 3), we again found a larger difference in the summary score between both versions in the group of patients with considerable health burden (− 4.8, 95% CI − 6.9 to − 2.8, *p* < 0.001, global QoL < 50, *n* = 57) compared to patients with lower/no health burden (− 1.4, 95% CI − 2.6 to − 0.2, *p* = 0.022, global QoL ≥ 50, *n* = 172). Furthermore, 7 out of 14 scale differences in the higher health burden group were statistically significant and all differences exceeded the 5 score point criterion.

In addition to the comparison of the two EORTC QLQ-C30 versions, the study design further allows for the comparison between paper-based and computer-based assessment of the questionnaire. Subgroup analyses revealed that differences between the both versions were more pronounced in the computer-based version than in the paper-based version (Table 3). However, the 5 score point criterion was only exceeded in the between-group comparison within the computer-based assessment.

### Study 2

German respondents were randomly assigned either to the conventional EORTC QLQ-C30 questionnaire version 3.0 (response option 3 = *mäßig*, *n* = 1006) or the optimized version (response option 3 = *ziemlich*, *n* = 1027).

Participants in study 2 comprised of a representative sample of the German general population surveyed in the context of a large-scale international online norm data survey [9]. As shown in Table 4, the median age was 54 years, 50% were female and most participants (58%) reported at least one disease.

**Table 4:** Study 2: sample characteristics

German population (*N* = 2033)	
Age in years m (SD); med (IQR); min–max	53.7 (15.0), 54.0 (42.5–66.0), 18–90
Sex no. (%)	
Female	1012 (49.8)
Male	1021 (50.2)
Education no. (%)	
Less than some post compulsory education	237 (11.7)
At least some post compulsory (~ upper secondary) education	1773 (87.2)
Missing	23 (1.1)
Country no. (%)	
Germany	2,033 (100)
Health condition no. (%)	
No disease	715 (35.2)
At least one disease	1182 (58.1)
Prefer not to answer/unclear answer	136 (6.7)
Multiple answers possible > 100%	
Chronic pain	551 (27.1)
Heart disease	168 (8.3)
Cancer (excluding basal cell carcinoma)	66 (3.2)
Depression	181 (8.9)
Chronic obstructive pulmonary disease	65 (3.2)
Arthritis	305 (15.0)
Diabetes	232 (11.4)
Asthma	115 (5.7)
Anxiety disorder	86 (4.2)
Obesity	175 (8.6)
Drug/alcohol use disorder	20 (1.0)
Other	343 (16.9)

*m* mean, *SD* standard deviation, *med* median, *IQR* inter quartile range

As shown in Table 2, the unadjusted analysis showed a significantly higher summary score for the optimized EORTC QLQ-C30 version compared with the conventional EORTC QLQ-C30 version (m = 83.6, sd = 15.9 vs. m = 82.0, sd = 17.7; *p* = 0.038). Multivariable analyses adjusted for age, sex, and health burden yielded even stronger effects: the mean difference of the summary score was − 3.1 (95% CI − 4.6 to − 1.5; *p* < 0.001, Table 3), and 9 out of 14 single scales showed statistically significant differences, i.e., *p* values < 0.05. None of the observed differences reached 5 points or more (Table 3).

When taking a closer look at respondents with considerable health burden (*n* = 370, global QoL < 50) versus those with lower/no health burden (*n* = 1663, global QoL ≥ 50), the difference in the summary score between both versions was more pronounced in the high burden group (− 4.5, 95% CI − 7.3 to − 0.17, *p* = 0.002) than in the low burden group (− 1.6, 95% CI − 2.9 to − 0.3, *p* = 0.016, Table 3). In the higher health burden group, 8 out of 14 differences in single scales were statistically significant, and 7 of these differences exceeded the 5 point criterion.

Significant and minimally important differences between conventional and optimized EORTC QLQ-C30 versions are summarized up in Additional file 1: Appendix Table S3.

### Choice of response options in the *mäßig* and in the *ziemlich* questionnaire versions (Studies 1 and 2)

**Fig. 1:**
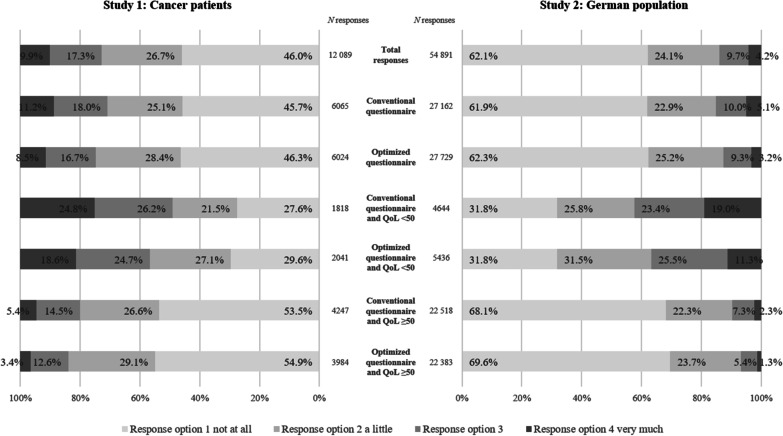
Frequencies of chosen response option—German population and cancer patients’ first assessment. The EORTC QLQ-C30 questionnaire was presented in two versions. The conventional questionnaire used *mäßig* and the optimized version used *ziemlich* as response option 3 (*quite a bit)* of the 4-point Likert scale. Responses to each response option (1–4) are presented for the total sample and are further separated for (1) subjects with QoL < 50 and QoL ≥ 50 as well as for (2) questionnaire version with response option *mäßig* and questionnaire version with response option *ziemlich*. German population: A total of N = 54,891 responses were given from N = 2033 respondents to items 1–27 (no missing responses). Cancer patients: At first assessment, a total of *N* = 12 089 responses were given from *N* = 450 patients to items 1–27 (missing responses *n* = 61 [0.5%])

We collapsed the total number of responses for response options 1, 2, 3 and 4 across the 27 items that made up the summary score and compared their distributions between the questionnaire versions in studies 1 and 2 (Fig. 1 and Table 5).

**Table 5:** Study 1: changes in frequencies of chosen response option—cancer patients

	Optimized questionnaire (*ziemlich)*				Total
	1 not at all	2 a little	3 quite a bit (*ziemlich)*	4 very much	
*Conventional questionnaire (mäßig)*					
1 not at all					
*n*	**2628**	447	59	10	3144
%	**83.6%**	14.2%	1.9%	0.3%	100%
2 a little					
*n*	433	**950**	177	22	1582
%	27.4%	**60.1%**	11.2%	1.4%	100%
3 quite a bit (*mäßig*)					
*n*	83	392	**438**	57	970
%	8.6%	40.4%	**45.2%**	5.9%	100%
4 very much					
*n*	15	58	178	**202**	453
%	3.3%	12.8%	39.3%	**44.6%**	100%
Total					
*n*	3159	1847	852	291	6149
%	51.4%	30.0%	13.9%	4.7%	100%

The EORTC QLQ-C30 was presented in two versions. The conventional version used *mäßig* and the optimized version used *ziemlich* as response option 3 of the 4-point Likert scale

A total of *N* = 229 patients responded to both questionnaire versions and reported no changes in health as well as quality of life between both assessments. A total of *N* = 6149 responses to items 1–27 (missing responses *n* = 34 [0.5%]) were gained. Responses to each response option (1–4) are presented for the total sample. Bold numbers indicate no change in chosen response option between both questionnaire versions

Looking at Study 2 and analyzing responses (*N* = 54,891) of all respondents (*N* = 2033) (Fig. 1), it appeared that frequencies in response option 1 (*überhaupt nicht* [*not at all*]) were practically identical in the *mäßig* and *ziemlich* versions (61.9% and 62.3%, respectively). However, the introduction of the term *ziemlich* modified the meaning of the entire scale and consequently the choice of the remaining response options 2, 3, and 4. Firstly, as expected, the response option 4 (*very much*) was used more frequently in the *mäßig* version than in the *ziemlich* version 5.1% versus 3.2%. Secondly, the difference between the percentage of respondents choosing options 2 and 3 was 12.9% in the *mäßig* version, and 15.9% in the *ziemlich* version.

These two effects were particularly pronounced in respondents with a poor general health status (global QoL < 50). While 19% percent of these respondents chose the highest response option 4 (*very much)* in the *mäßig* condition, only 11.3% chose this response option in the *ziemlich* condition. Furthermore, in the *ziemlich* version, response options 2 and 3 were more distinct (6.0% difference) than in the *mäßig* condition, showing a 2.4% difference.

Comparable effects were obtained in the first assessment sample of study 1 (Fig. 1).

Further analyses included cancer patients who answered both versions consecutively and reported no health changes between the two assessments (*n* = 229, Table 5). While there was a high overlap of 83.6% in choosing response option 1 (*not at all*) across the two versions, overlap for the other 3 response options was considerably lower, i.e., 60.1%, 45.2%, 44.6%, respectively.

That is, 39.3% of respondents who chose response option 4 (*very much*) in the *mäßig-*version switched to option 3 (= *quite a bit*) in the *ziemlich*-version (Table 5). This effect was particularly pronounced in patients with good health (QoL ≥ 50) who switched in 43.5% of the cases, whereas this percentage was only 37.0% in patients with higher health burden (QoL < 50) (data not shown).

## Discussion

Based on the observation that response options are not equidistant in the German version of the EORTC QLQ-C30, the main aim of this research was to test the hypothesis that the current German response option 3 is suboptimal and may bias results towards the worse end of the scale, i.e., worse/lower functioning and higher symptoms.

As hypothesized, the main finding of the present studies is that the optimized EORTC QLQ-C30 version yielded slightly lower symptom and higher functioning scores. The magnitude of mean differences in adjusted multivariable analyses was 4.5 (cancer patients, between-group comparison, *n* = 450), 3.1 (cancer patients, within-group comparison, *n* = 229), and 3.1 (German reference sample, *n* = 2033). This effect became particularly pronounced when we had a closer look at respondents with a high health burden: 6.8, 4.8, and 4.5 mean difference in score points, respectively. These values are at the lower end of Osoba’s widely cited 5–10 point difference criterion for minimal important clinical changes on the EORTC QoL scales [13]. These effects were not only obtained for the summary scale, but also for numerous of the single scales of the EORTC QLQ-C30. The scale that showed the highest proportion of significant differences was physical functioning, followed by appetite loss, role functioning, emotional functioning, and fatigue.

This effect can be interpreted through a psychological theory which posits that scaling labels are of informational value for respondents, guiding them to understand the question and to elicit the most “appropriate” answer in a given context [5, 6]. In the *mäßig-*version, *mäßig* (response option 3) is semantically very close to response option 2 (*wenig* = *a little),* but considerably far apart from response option 4 (*sehr* = *very much)*. Therefore, respondents may have problems to differentiate between *wenig* and *mäßig* and have an inclination to choose *sehr* (*very much*), particularly when they suffer from an impaired health status. Introducing *ziemlich* changed the entire response environment, as it lies more equally balanced between response options 2 (*a little*) and 4 (*very much*). Thus, the response options have a clearer meaning, now rendering *ziemlich* (*quite a bit*) a worthwhile option in the case of health problems and making *sehr* (*very much*) less attractive.

This interpretation is in line with the pooled frequencies of each of the four response options across 27 questionnaire items. We saw that the differences in frequencies between *mäßig* and *wenig* are less pronounced than between *wenig* and *ziemlich*. Furthermore, for respondents with high health burden, *sehr* (*very much*) was regularly an appropriate response option in the *mäßig*-version, and much more so than in the *ziemlich*-version where *ziemlich* was still considered an adequate reflection of their perceived health status.

Furthermore, we investigated the possibility of potential differences between the paper-based and the computer-based assessment. In the computer-based assessment each item is presented individually at the screen together with the response labels, whereas in the paper version the response labels are shown only at the very beginning of the questionnaire. There is reason to believe that these differences in the presentation format may amplify the wording effect, and this effect becomes more pronounced in the computer-based assessment. We found some indication for this sort of amplification, but it was not as strong and as consistent as one might expect.

Adopting a broader perspective outside the peculiarities of response labels in specific language versions (in this case German), the implications of this project are twofold.

Firstly, this project is a good example of how quality assurance can be done in the field of patient-reported outcomes instruments. To date, only few examples have been published in this area. Quality assurance projects have focused on paper-based versus electronic assessment (particularly migration of the former to the latter) [21], translation and linguistic validation [2], or compliance with regulators’ (FDA, EMA) perspectives on outcome assessment [22, 23]. We are not aware of a study like this that systematically called into question existing response options and made a head-to-head comparison between two questionnaire versions.

Secondly, this project is also a timely reminder that psychological processes play a crucial role in QoL assessment. QoL research is preoccupied with psychometrics, statistical models, and technical details, at the expense of analyzing the dynamics underlying the interplay between the responder and the questionnaire. In order to understand and interpret answers to questionnaires correctly, a thorough analysis of the cognitive and emotional underpinnings is essential. Ultimately, questionnaires are communication tools that are of value only if the questionnaire developer, the sender (i.e., the patient) and the receiver (i.e., the researcher or clinician) of the information are on the same page.

Limitations of the study may relate to the use of the EORTC QLQ-C30 summary score. An argument can be reasonably made, that this summary score is composed of many and diverse QoL aspects rendering it difficult to interprete and thus, meaningless for use in clinical studies. In fact, many clinical studies are often based on well-defined hypotheses and therefore focus on specific QoL scales or side effects. A strength of the summary score comes into play, when a hypothesis with regard to a specific scale is not at the core: it avoids problems connected with exploratory multiple statistical testing of numerous QoL scales (“p-hacking”). This property motivated the creation of the summary score in the first place and this was also a reason why we made use of it. We expected to see differences between the two questionnaire versions without being able to specify beforehand which of the available 14 single scales would show the hypothesized effects. Therefore, our analysis strategy was to have a look at the summary scale first, and only in case of a significant effect, the single scales were explored further. It should be noted that the EORTC Quality of Life Group is in the process of exploring the potential of the summary score and is about to prepare a guideline on its use.

A further limitation of the present analyses lies in their exclusive use of methods of classical test theory. We acknowledge the conceptual and statistical superiority of item response theory (IRT), which is used by the EORTC Quality of Life Group particularly in the construction of item sets for computer adaptive testing [24]. To obtain reliable results, IRT analyses require larger sample sizes than were available here. Additional studies focusing on the measurement properties of the updated questionnaire including a wider range of methodological approaches are desirable.

## Conclusion

Our starting point was that the German translation of the *quite a bit* response category was not located at the right place according to the assumption of equidistance. This pair of studies tested a revised response option, confirming that the revised version solves the problem, and should therefore be used in the future.

## Supplementary Information

**Additional file 1**. **Appendix Table S1.** Summary of the main results of the experimental response option studies. **Appendix** Basic psychometric properties. **Appendix Table S2.** Internal consistency, convergent and discriminant validity for multi-item scales of the EORTC QLQ-C30 conventional and optimized questionnaire version. **Appendix Table S3.** Summary of significant and minimally important differences between QLQ-C30 versions across all analyses. **Appendix Figure S1.** Design of study 1.

## Data Availability

The datasets used and/or analysed during the current study are available from the corresponding author on reasonable request.

## Abbreviations

ANCOVA: Analysis of covariance
EORTC QLQ-C30: European Organization for Research and Treatment of Cancer Quality of Life Questionnaire-Core
Fig: Figure
GfK SE: Growth from Knowledge Societas Europaea
IQR: Interquartile range
MOA: Mode of assessment
QoL: Quality of life
95% CI: 95% Confidence interval
