# Supplementary material for: Investigating the response scale of the EORTC QLQ-C30 in German cancer patients and a population survey
Source: Health Qual Life Outcomes. 2021 Oct 9;19:235. doi: 10.1186/s12955-021-01866-x (PMC8501673; doi:10.1186/s12955-021-01866-x)
Supplement: Supplementary file 1 — Additional file 1. Appendix Table S1. Summary of the main results of the experimental response option studies. Appendix Basic psychometric properties. Appendix Table S2. Internal consistency, convergent and discriminant validity for multi-item scales of the EORTC QLQ-C30 conventional and optimized questionnaire version. Appendix Table S3. Summary of significant and minimally important differences between QLQ-C30 versions across all analyses. Appendix Figure S1. Design of study 1. [file 12955_2021_1866_MOESM1_ESM.docx]

**Appendix**

**Table S1 Summary of the main results of the experimental response option studies^1^**

|  |  | Intensity of suggested term | | Among the top three in terms of appropriateness |
| --- | --- | --- | --- | --- |
|  | ***N*** | **mean**^2^ | **sd** |  |
| *Mäßig* |  |  |  |  |
| Study 1 | 111 | 45.63 | 13.69 | No |
| Study 2 | 83 | 38.49 | 13.66 | No |
| Study 3 | 140 | 41.42 | 13.65 | Yes |
| *Ziemlich* |  |  |  |  |
| Study 1 | 111 | 71.44 | 13.46 | Yes |
| Study 2 | 83 | 69.41 | 13.56 | Yes |
| Study 3 | 140 | 71.25 | 15.19 | Yes |

^1^Source: *Investigating the German response scale of the European Organisation for Research and Treatment of Cancer Quality of Life Questionnaire (EORTC QLQ-C30) – a three-step approach*. Interim report on Step I – June 2015 by Michael Koller and Karolina Müller (Internal technical paper).

Study 1 involved 111 students (mean age 21.6 years, sd = 2.74, range 18 to 34; 84.7% females) who filled in a list of 22 response alternatives.

Study 2 involved 83 students (mean age 20.8 years, sd = 2.74, range 18 to 36; 64.8% females)

who filled in a shortened list of 7 response alternatives.

Study 3 involved 140 respondents (76 cancer patients and 64 controls; 66.4% females) who filled in a final list of 5 response alternatives.

^2^ideal value = 67 on a scale ranging from 0 to 100

**Basic psychometric properties**

According to classical test theory, basic psychometric tests were calculated to explore the psychometric performance of both EORTC QLQ-C30 versions. Cronbach’s alphas for multi-item scales were computed separately in cancer patients (Study 1) and the German general population (Study 2). By convention, alpha values ≥ .70 are considered as acceptable indicators for a scale’s internal consistency ^17^. Item-scale correlations were used to determine convergent (r ≥ .40; corrected for overlap) and discriminant (r < .40) validity. A definite scaling error existed if an item correlated significantly less with its own scale than with another scale ^18,19^.

Results of the basic psychometric tests on the EORTC QLQ-C30 multi-item scales for the conventional (*mäßig*) version and the optimized (*ziemlich*) version are shown in Table 2. Cronbach’s alphas of multi-item scales were comparably high in both versions, exceeding the .70 threshold in both versions (with the exception of nausea/vomiting and cognitive functioning on some assessments). Correlation coefficients supported convergent and discriminant validity, and no scaling errors occurred in either of the two versions across study 1 and 2. Thus, the proposed scale structure of the conventional and optimized EORTC QLQ-C30 version is supported.

**Table S2 Internal consistency, convergent and discriminant validity for multi-item scales of the EORTC QLQ-C30 conventional and optimized questionnaire version**

|  | | ***mäßig* version** | | | | ***ziemlich* version** | | | |
| --- | --- | --- | --- | --- | --- | --- | --- | --- | --- |
|  | Item # | Cronbachs Alpha | Convergent validity | Discriminant validity | Scaling error | Cronbachs Alpha | Convergent validity | Discriminant validity | Scaling error |
| **Cancer patients *N* = 450** | | ***n* = 423^1^** | | | | ***n* = 426^1^** | | | |
| Physical Functioning | 1 to 5 | .86 | .49 to .78 | .15 to .65 | 0 | .86 | .51 to .80 | .09 to .71 | 0 |
| Role Functioning | 6, 7 | .89 | .80 | .19 to .63 | 0 | .90 | .83 | .23 to .69 | 0 |
| Emotional Functioning | 21 to 24 | .84 | .58 to .72 | .16 to .66 | 0 | .83 | .58 to .70 | .12 to .64 | 0 |
| Cognitive Functioning | 20, 25 | .71 | .56 | .10 to .58 | 0 | .69 | .53 | .11 to .60 | 0 |
| Social Functioning | 26, 27 | .82 | .69 | .20 to .57 | 0 | .84 | .72 | .17 to .60 | 0 |
| Global Health Status / QoL | 29, 30 | .92 | .85 | .15 to .63 | 0 | .92 | .86 | .20 to .62 | 0 |
| Fatigue | 10, 12, 18 | .88 | .75 to .80 | .19 to .67 | 0 | .89 | .77 to .82 | .22 to .76 | 0 |
| Nausea and Vomiting | 14, 15 | .64 | .52 | .12 to .53 | 0 | .64 | .51 | .12 to .48 | 0 |
| Pain | 9, 19 | .83 | .71 | .11 to .56 | 0 | .84 | .73 | .14 to .59 | 0 |
| **German population *N* = 2033** | | ***n* = 1006** | | | | ***n* = 1027** | | | |
| Physical Functioning | 1 to 5 | .85 | .50 to .79 | .21 to .69 | 0 | .87 | .51 to .78 | .24 to .71 | 0 |
| Role Functioning | 6, 7 | .87 | .76 | .29 to .74 | 0 | .91 | .77 | .28 to .76 | 0 |
| Emotional Functioning | 21 to 24 | .86 | .63 to .76 | .23 to .61 | 0 | .84 | .68 to .78 | .26 to .65 | 0 |
| Cognitive Functioning | 20, 25 | .76 | .61 | .32 to .60 | 0 | .72 | .64 | .27 to .55 | 0 |
| Social Functioning | 26, 27 | .90 | .81 | .35 to .61 | 0 | .85 | .76 | .34 to .64 | 0 |
| Global Health Status / QoL | 29, 30 | .91 | .83 | .22 to .66 | 0 | .93 | .85 | .26 to .68 | 0 |
| Fatigue | 10, 12, 18 | .88 | .74 to .79 | .27 to .66 | 0 | .90 | .73 to .76 | .29 to .66 | 0 |
| Nausea and Vomiting | 14, 15 | .81 | .69 | .18 to .60 | 0 | .63 | .72 | .19 to .59 | 0 |
| Pain | 9, 19 | .92 | .86 | .27 to .68 | 0 | .86 | .82 | .29 to .72 | 0 |

The EORTC QLQ-C30 questionnaire was presented in two versions. The conventional questionnaire used *mäßig* for response option 3 (*quite a bit*), and the optimized version used *ziemlich*.

Convergent validity: item-scale correlations with own scale corrected for overlap. Discriminant validity: item-scale correlations with other scales. Scaling error: number of definitive scaling errors, i.e., cases in which an item was significantly higher correlated with another scale.

^1^Sample size varies due to missing values in cancer patients.

**Table S3 Summary of significant and minimally important differences between QLQ-C30 versions across all analyses**

|  |  | **Univariable analyses** | | | **Multivariable analyses** | | | | | | | | |
| --- | --- | --- | --- | --- | --- | --- | --- | --- | --- | --- | --- | --- | --- |
|  | Proportion of significant differences  per scale | Cancer patients (between-group) | Cancer patients (within-group) | German population | Cancer patients (between-group) | Cancer patients (between-group)  higher health burden  (QoL<50) | Cancer patients (between-group)  lower health burden  (QoL≥50) | Cancer patients (within-group) | Cancer patients (within-group)  higher health burden  (QoL<50) | Cancer patients (within-group)  lower health burden  (QoL≥50) | German population | German population  (  higher health burden  (QoL<50) | German population  ()  lower health burden  (QoL≥50) |
| ***n*** |  | *450* | *229* | *2033* | *450* | *144* | *306* | *229* | *57* | *172* | *2033* | *370* | *1663* |
| **Summary Score** | 10/12 |  | * | * | * | * |  | * | * | * | * | * | * |
| **PF** | 8/10^1^ |  | * | * |  |  |  | * | * | * | * | * | * |
| **RF** | 5/10 |  | * |  |  |  |  | * | * |  | * | * |  |
| **EF** | 5/10 |  | * |  | * | * |  | * |  | * |  |  |  |
| **CF** | 3/10 |  |  |  | * |  |  |  |  |  | * | * |  |
| **SF** | 4/10 |  |  |  |  |  |  | * | * |  | * | * |  |
| **FA** | 5/10 |  |  |  |  |  |  | * | * |  | * | * | * |
| **NV** | 0/10 |  |  |  |  |  |  |  |  |  |  |  |  |
| **PA** | 4/10 |  |  |  |  |  |  | * | * |  | * |  | * |
| **DY** | 4/10 |  |  |  |  |  |  | * | * |  | * | * |  |
| **SL** | 4/10 |  | * |  | * |  |  | * |  | * |  |  |  |
| **AP** | 6/10 |  |  |  | * | * |  | * | * |  | * | * |  |
| **CO** | 0/10 |  |  |  |  |  |  |  |  |  |  |  |  |
| **DI** | 2/10 |  |  |  |  | * |  |  |  |  |  |  | * |
| **FI** | 4/10 |  |  |  |  | * |  | * |  |  | * | * |  |
| Proportion of significant scale differences per sub-group specific analysis | | - | 4/14 | 1/14 | 4/14 | 4/14 | - | 10/14 | 7/14 | 3/14 | 9/14 | 8/14 | 4/14 |

*denotes a statistically significant difference (p < 0.05). Statistically significant differences for scales were only presented if the Summary Score differed significantly. Cells with grey shades denote a minimally importance differences of 5 score points or more ^14^.

PF= Physical Functioning, RF = Role Functioning, EF = Emotional Functioning, CF = Cognitive Functioning, SF = Social Functioning, FA = Fatigue, NV = Nausea and vomiting, PA = Pain, DY = Dyspnea, SL = Insomnia, AP = Appetite loss, CO = Constipation, DI = Diarrhea, FI = Financial difficulties

^1^The denominator relates to the number of statistically significant effects of the Summary Score.

**Figure S1 Design of study 1**

The EORTC QLQ-C30 questionnaire was presented in two versions. The conventional questionnaire used *mäßig* for response option 3 (*quite a bit*), and the optimized version used *ziemlich*.

To ensure a high participation rate in test-retest analyses, patients within the paper-based assessment arm received an addressed and stamped envelope with the second questionnaire including the due date on which the questionnaire had to be filled out. Within the electronic assessment arm, the time between both assessments was chosen so that methodological aspects and patient needs were met. For methodological aspects, the time frame should be long enough to exclude memory effects and short enough to exclude real changes in quality of life. To control for real changes in quality of life, anchor questions were provided. If possible, the second assessment was scheduled in accordance with patients’ appointments at the clinic site.

Of the 450 included patients, 404 patients (90%) filled out the questionnaire twice. Accidentally, four out of the 404 patients filled out the same questionnaire twice and were not included in the analyses comparing the two questionnaire versions.

Of the 46 patients without second assessment, 28 patients were in the paper-based assessment arm and no reason for non-compliance can be given. Of the 18 patients in the electronic assessment arm, the second assessment was not done due to the following reasons: decline to participate (n = 3), significant deterioration of health or mental fitness (n = 2), patient missed the second appointment or the investigator missed the patient at second appointment (n = 8), and for five patients no information was provided.
